# A Review on the Rising Prevalence of International Standards: Threats or Opportunities for the Agri-Food Produce Sector in Developing Countries, with a Focus on Examples from the MENA Region

**DOI:** 10.3390/foods7030033

**Published:** 2018-03-03

**Authors:** Dima Faour-Klingbeil, Ewen C. D. Todd

**Affiliations:** 1 DFK for Safe Food Environment, 30559 Hannover, Germany; 2 Ewen Todd Consulting, Okemos, MI 48864, USA; todde@msu.edu

**Keywords:** food safety standards, fresh produce, agri-food, good agricultural practices, the Middle East and North Africa Region, global trade, the Food Safety Modernization Act

## Abstract

Food safety standards are a necessity to protect consumers’ health in today’s growing global food trade. A number of studies have suggested safety standards can interrupt trade, bringing financial and technical burdens on small as well as large agri-food producers in developing countries. Other examples have shown that economical extension, key intermediaries, and funded initiatives have substantially enhanced the capacities of growers in some countries of the Middle East and North Africa (MENA) region to meet the food safety and quality requirements, and improve their access to international markets. These endeavors often compensate for the weak regulatory framework, but do not offer a sustainable solution. There is a big gap in the food safety level and control systems between countries in the MENA region and those in the developed nations. This certainly has implications for the safety of fresh produce and agricultural practices, which hinders any progress in their international food trade. To overcome the barriers of legal and private standards, food safety should be a national priority for sustainable agricultural development in the MENA countries. Local governments have a primary role in adopting the vision for developing and facilitating the implementation of their national Good Agricultural Practices (GAP) standards that are consistent with the international requirements and adapted to local policies and environment. Together, the public and private sector’s support are instrumental to deliver the skills and infrastructure needed for leveraging the safety and quality level of the agri-food chain.

## 1. Introduction

Despite decades of research, the enactment of food laws, stricter regulations, and enforcement in both developed and developing countries, foodborne illnesses still persist as a global public health issue [1]. Although many foodborne disease outbreaks are of unknown origin, increasing numbers are traced to both well-established and newly emerged pathogens [2]. The current report of the WHO (World Health Organization) estimates of the global burden of foodborne diseases recognized 31 hazards (bacteria, viruses, parasites, toxins, and chemicals) that have caused 22 diseases [1]. The most frequent causes of foodborne illness were diarrheal disease agents, particularly pathogenic *Escherichia coli* (*E. coli*), norovirus, *Campylobacter*, and non-typhoidal *Salmonella*. These were responsible for 70% of the burden of foodborne disease. African and Southeast Asian regions have the highest foodborne disease incidence and the highest death rates, followed by the Eastern Mediterranean Region [1].

One major concern in the last two decades, in many parts of the world, is the recognition of fresh produce as vehicles in during foodborne disease outbreaks [2,3,4]. Between 2011 and 2013, there were 170 alert notifications by the Rapid Alert System for Food and Feed (RASFF) in the European Union (EU) concerning pathogenic microorganisms in fruits and vegetables [5]. According to the Centre for Science in the Public Interest (CSPI)’s database, fresh produce that are often eaten raw cause more foodborne illness than any other single category of food in the United States (US) [6]. The review of foodborne illnesses in the US from 2004–2013 showed that cucumbers, pepper, and cilantro continued to cause illnesses, and that many outbreaks would remain unsolved and their origins untraced [6]. However, investigations on 11 farms in Mexico, which eventually linked cyclosporiasis outbreaks caused by cilantro from 2012 to 2015 to Mexican fields in Puebla, showed that strains in the fields originated from human feces [6]. This root cause analysis led to the U.S. Food and Drug Administration (USFDA) banning imports from Mexico until better documentation on growing and harvesting practices in that country was available. Overall, the US and EU have reported a total of 377 and 198 produce-associated outbreaks, respectively, for the period 2004–2012 [7]. Nonetheless, even these high numbers are thought to be sporadic cases linked to the consumption of produce [8]. 

At each step of a food chain, produce is subjected to a range of growing, harvesting, processing, and storage conditions. These functions along the food chain are becoming increasingly developed, as marketing is allowing the availability of produce and other agri-food supplies to consumers year-round in a fresh state [9]. However, high-volume production magnifies the risk of foodborne illness, as in the case of the 2006 spinach outbreak in California [10]. Also, global food sourcing can introduce food safety risks, which are often amplified through the international food chain. Many *E. coli* O157 and other verotoxigenic (VTEC) or shigatoxigenic (STEC) serotype outbreaks have been linked to fresh produce. The largest was the *E. coli* O104 outbreak in Germany and 15 other countries in 2011, with over 3900 people affected, and 800 persons suffering from hemolytic uremic syndrome, 53 of whom died; most cases (3816) and deaths (51) were in Germany [11]. 

The *E. coli* O104 strain showed an unusual combination of virulence factors of STEC and enteroaggregative *E. coli* (EAggEC). Initially, Spanish cucumbers imported from Spain were thought to be the source of the outbreak by the German health authorities, because four cucumbers, including three imported from Spain, were carrying *Enterohaemorrhagic E. coli* (EHEC), but later the investigation recognised that Spanish greenhouse-grown cucumber samples did not contain the specific responsible *E. coli* variant. This embargo caused economic hardship, with losses of US Dollar $200 million per week to Spanish fresh produce exporters [12]. Other countries warned their populations to avoid produce items, and there were some other trade embargoes. 

Tracing forward and backward the dissemination of the implicated sprouting seeds demonstrated that all of the outbreak clusters, for which there was sufficient information, could be linked to a single sprouted seed producer in Germany, who imported fenugreek seed. However, fenugreek sprouts were mostly sold as mixtures of sprouts, complicating the investigation. The investigators then pinpointed that a specific lot of fenugreek seeds imported from Egypt was the most likely source, although no samples of the seed tested positive. Also, it was possible that other lots from the same exporter and importer could also be implicated. In July 2011, the EU banned the import of certain Egyptian seeds and beans until at least October [13]. Trace-back investigation showed that the fenugreek seeds were shipped to Antwerp, Belgium, then trucked into Germany to an unidentified importer, some of which was resold to the German sprout producer. Other seeds were shipped to France as garden seed packages. Illnesses in both Germany and France were linked to these seeds. Therefore, the German authorities considered all lots of fenugreek from the Egyptian exporter as suspect, assuming that fecal contamination of the seeds likely occurred during their production or distribution in Egypt, and the shelf life of the seed can be up to five years. The EU ban was extended until 2012, after an audit showed that measures taken by the Egyptian authorities were deemed insufficient to reduce the risks [14]. This episode illustrates the extent of emerging microbial risks through the globalized food trade with produce, and the difficulties for regulators to address them. Thus, because much of produce is eaten fresh, with no final kill step, regulators are trying to address such threats to public health through the adoption of prevention rather than control practices [15].

However, typically, major changes in legislation and updating regulations are largely influenced by food scares, where the public expresses concern for the food safety oversight system. There were also continuing outbreaks starting in the 1980s of *Listeria monocytogenes* (*L. monocytogenes*), *E. coli* O157, and *Campylobacter*, not just from meats, but also from produce. The unexpected bovine spongiform encephalopathy (BSE) episodes in the 1990s, commonly known as “mad cow disease”, although initially discovered in the United Kingdom (UK), quickly affected many parts of the world because of trade in livestock. The impact of this epidemic was catastrophic, with 170,000 cattle being directly affected and 4.4 million killed as a precaution against further spread of the prion; most devastating were the deaths of 164 people to date in Britain, from New Variant Creutzfeld-Jacob disease (vCJD), the human form of BSE. Additional public concerns arose over the production and sale of milk derived from bovine somatotropin, pesticide use in crops, and increasing antibiotic resistance in humans and animals. This transformed food safety, being viewed by many as not just a scientific matter but also a highly political issue. The EU legislation also went stricter, by banning the use of some antibiotics as animal feed additives and growth promoters in farm animals [16]. Such crises instigated the process of harmonization of food legislation among the EU countries and EU commercial partners. The European Parliament is now prioritising the consumer at the economic heart of the single market. Examples of the politicians’ reactions to these food scares are the Food Standards Agency (FSA) in the UK in 2000 and the European Food Safety Authority (EFSA) for the EU in 2003. These were created with a clear focus on the consumer, as opposed to the product or market [17,18]. The US completely redesigned its food safety oversight through the Food Safety Modernization Act (FSMA) of 2011, and many technical regulations on food safety, plant protection, and labelling were developed by national governments and the private sector [19]. 

There is no doubt that these series of frequent food outbreaks, the complexity of the global food chain, and consumers’ demand for high-quality foods in high-income countries, whatever the source, gave rise to the proliferation and strengthening of food and agricultural quality standards over the last two decades. However, the tightening control on imported goods and the requirements for the application of a preventive approach from farm to market still pose huge burdens on most exporting developing countries, making food trade more complicated [20]. 

For some developing countries, the fact that food safety is not a national priority hinders the transition towards the implementation of food safety regulations [21]. From the regulatory perspective, some countries have not yet established adequate surveillance or reporting mechanisms to identify hazards and track foodborne diseases, in order to fully evaluate their food safety problems, including those involving produce, and ensure risk management along the complete value chain [22]. For instance, PulseNet Middle East was established in 2006 to support the food safety regional plan and promote technical collaboration between countries, following a consultation meeting held at the WHO Eastern Mediterranean Regional Office (EMRO) in Cairo, Egypt. Nonetheless, even in countries with reporting systems in place, there is still underreporting and limited disease surveillance systems [23]. This explains the assertion of some developing countries that the safety of produce is not a specific source of concern to them [24]; hence, the rare monitoring of fresh produce-related diseases and the limited recognition of their burden [25]. Eventually, these countries lack the capability to meet the World Trade Organization (WTO) requirements and to verify compliance to international standards throughout the value chain [26,27], which is a common case in most countries of the Middle East and North Africa (MENA) region [28]. 

At the national level, the impact of the growing prevalence of standards on the international trade of agri-foods in developing countries, particularly in the MENA region, has not previously been sufficiently discussed. This paper reviews the growing concerns over fresh produce safety, the frequent incidences of food outbreaks linked to its consumption, and the current trends in national and international standards to reduce the spread of risks in the global food chain market. The challenges and disparities in the food safety governance between developed and developing countries, burdens of compliance, and discerning aspects of integrated agri-food chains were examined. Given that the EU and US are major importers of food and agri-food products from developing countries [29], this review is centered around the EU regulations and FSMA rule requirements. 

Therefore, this paper provides a synthesized overview of current evidence on the prevalent role of food safety standards as a catalyst or a hurdle to food trade, in order to create an understanding on intervening factors and their influence on the agri-food export activities in the MENA countries.

## 2. Sources of Data

Searches of literature were done using web-based search engines (i.e., Google and Google Scholar, as well as research databases, such as PubMed, Science Direct, and Web of Science). Keywords and search phrases were used to identify materials that describe the research topic. The topics of interest for review were categorized as:

- Fresh produce-associated food outbreaks, fresh produce contamination
- The evolution of the food safety standards, in terms of applications and development
- The different types of food safety standards
- The international regulatory framework for food safety
- Fresh produce safety, requirements, and guidelines (with search focus on the EU and US)
- Challenges in food safety control systems in the MENA countries
- Data on rejected goods from the MENA countries and import ban
- Impact of food safety standards on fresh produce trade
- Integrated agri-food chains, in the context of standards implementation and food export programs

The search was limited to original peer-reviewed scientific papers, scholarly books, statistical information, and grey literature (i.e., reports of regional and national workshops or meetings, as well as governmental and non-governmental reports, such as from the Food and Agriculture Organization of the United Nations (FAO), WHO, EFSA, or the World Bank). Studies and reports related to economic analysis of food value chains were excluded. To overcome the scant literature on the MENA countries, media coverage and local journals were included as a source of data. Repetitive coverage of issues was excluded. Additionally, the search was expanded to include studies on agri-food safety and compliance models in developing countries outside the MENA region. 

## 3. Food Safety Concerns and Standards Are Growing Globally

Today, many consumers have a wide array of food products that are sourced from various parts of the world, e.g., China, the Middle East, Asia, India, and others. An increase of around 400 percent in the international trade in food has been recorded from 1989 to 2000, and the fastest growth in trade was observed among the high-value perishable products, including fresh fruits and vegetables [30]. However, the growth in volume and complexity continues to date, with a recorded growth of 38% of the world production of fresh produce in the last decade [31]. The US reliance on imported food increased from 9% in 2000 to 16% of all food consumed in 2011. The imports account for nearly two-thirds of the fruits and vegetables and 80% of seafood eaten domestically in the US, and this is expected to increase by 10% annually with regards to Chinese imported foods [32]. 

The global trade of fresh produce is primarily driven by increased demands of a growing population, improved agricultural production technology, and changes in consumers’ dietary habits. This fact, combined with the involvement of many chain actors from different geographic regions in the interaction of global and local value chains, can introduce multiple new opportunities for food to become contaminated with harmful bacteria, viruses, parasites, or chemicals, especially when food is sourced from less developed countries or countries with lower food safety standards [33]. As an example, the US Department of Agriculture Economic Research Service notes that of food-related refusal reports, the three food industry groups with the most violations were vegetables (20.6% of total violations), fishery and seafood (20.1%), and fruits (11.7%). The reported violations included sanitary issues and pesticides, aside from technical issues related to unregistered processes in all three industry groups [34].

### 3.1. Fresh Produce-Related Foodborne Illnesses and Health Risks

The routes of contamination of fresh produce in the agricultural environment and down the food chain are numerous [35]. In general, pathogenic microorganisms can be transmitted to crops through contaminated seeds, raw or improperly composted manure, sewage-contaminated water used for irrigation, animals accessing crops or contaminated wash water, and via wild and domestic animals grazing on fields or water streams used in agriculture. The post-harvest treatment of fruits and vegetables, including handling, storage, transportation, and cleaning, may also lead to cross-contamination of the produce from other agricultural materials, or from the workers and food handlers [36,37,38,39]. As local risks can rapidly penetrate into international markets, the human exposure to a wide array of contaminants has increased, making transparency and traceability in the supply chains necessary, but also more complicated [9]. A wide spectrum of human pathogens have been associated with fresh vegetables, as indicated by many foodborne disease outbreaks; these include *E. coli* O104:H4, *L*. *monocytogenes*, *Salmonella*, viruses (hepatitis A virus, norovirus) and parasites (*Cryptosporidium parvum*, *Cyclospora cayetanensis*) [40,41]. *Salmonella enterica* and *E. coli* are the two major bacterial pathogens encountered in large outbreaks of foodborne illness associated with fresh produce, and have been traced to a wide variety of produce, including lettuce, salads, melons, sprouts, tomatoes, and many fruit- and vegetable-containing dishes (Table 1), with a low infectious dose of fewer than 40 viable cells for *E. coli* O157 [42]. Between 1990 and 2005, norovirus and *Salmonella* also emerged as common agents of produce-related outbreaks, followed by *E. coli*, at 40%, 18%, and 8%, respectively [43]. 

The rise in reported cases associated with produce has been attributed to increased awareness of the nutritional importance of the consumption of fresh vegetables, as a result of promotional campaigns for its health benefits [44]. But there has also been increased global trade of produce, improvements in outbreak investigations, more efficient detection methods in surveillance systems, and more awareness of investigators to consider raw vegetables as potential vectors of disease. For instance, the combination of DNA fingerprinting with well-developed epidemiological surveillance systems allows for more rapid detection of outbreak cases in widely-dispersed geographic regions. Although this is best illustrated in the developed world, developing countries are increasingly being encouraged to use better pathogen detection methodologies, along with improvements in agronomic practices [19,45]. Several recent incidences of outbreaks shed light on the growing trend in international trade of fresh produce and its contribution to outbreak occurrences [46,47,48,49], particularly from countries with lower food safety standards [2]. For instance, many cases of *E. coli* and *Salmonella* in the US have been associated with the consumption of imported as well as domestic foods, including fruit and vegetables [50]. 

### 3.2. The Rise in Prevalence of National and International Standards: Current Requirements and Risk Factors at the Primary Production Stage

The important principle of standards is the “preventive control” approach, which identifies the source of food safety hazards and develops mitigation strategies to eliminate or reduce the risks to acceptable levels before consumption, instead of relying on “end product” inspections. Standards can be voluntary or mandatory, private or public, and include product or process-related standards [57]. These standards can be specific to provide maximum acceptable levels related to pathogens or toxins such as contamination of aflatoxin or pesticides [58], but they can also be broader to include hygienic, sanitary, and phytosanitary measures issues. Apart from the creation of new agencies, national governments have responded to consumers’ concerns by imposing new legislation and regulations on the ground, to ensure safe and animal-friendly production, restricted pollution, and to economize the use of resources, e.g., the General Food Law (EU 2002/178) in Europe and the EU-BSE regulations. 

The priorities of national governments for the food supply are the preservation of their domestic markets and their consumers’ health, which in the developed world are both protected through advanced manufacturing and retailing systems, combined with government oversight [33]. The regulations may differ in rigor between countries, based on their health and trade policies, but generally it is accepted that the food industry, throughout the food chain, takes the primary responsibility for ensuring food safety and adhering to international standards. Accordingly, the fresh produce industry has witnessed a transition from food safety and quality control approaches, to the production of fresh produce with a food safety assurance and prevention approach to limit contamination [59]. These approaches require a system that is science-based and uses risk analysis to focus preventive efforts and risk management on the areas or processes that are most likely to cause foodborne illnesses [60]. 

Following the 2011 verocytotoxigenic *E.* *coli* (VTEC) O104 seed sprout outbreak in Germany [35], and a number of concurrent outbreaks of foods from non-animal origins in the EU, the assessment and ranking of public health risks posed by microbiologically-compromised fresh produce became paramount and essential for effective management [61]. As such, the EFSA, mandated by EC regulation 178/2002 to inform and assess the risks along the food chain, was asked to provide scientific opinions on the public health risk posed by pathogens on food of non-animal origin (FNAO). A total of six opinions have been issued on FNAO as of 2011, referring to the following food/pathogen combinations with a similar production system and identified as the most important risks within FNAO [24,62,63,64]:

1. The risk from VTEC in seeds and sprouted seeds (urgent request after VTEC crisis)
2. The risk from *Salmonella* and norovirus in leafy greens eaten raw as salads
3. The risk from *Salmonella* and norovirus in berries
4. The risk from *Salmonella* and norovirus in tomatoes
5. The risk from *Salmonella* in melons
6. The risk from *Salmonella*, *Yersinia*, *Shigella*, and norovirus in bulb and stem vegetables, as well as carrots

The agency also identified major risk factors at primary production during harvest and on-farm post-harvest, which are namely field history and adjacent land use, animal control (domestic and wild), water, manure, soil amendments, field management (including field sanitation and sanitary facilities), production activities, harvest activities, worker health, hygiene, sanitary facilities, and storage and distribution [24,65]. 

The EFSA recommendations verified the usefulness of the current provisions of the hygiene rules for foods of non-animal origin in the Regulation (EC) No. 852/2004. Under the hygiene rules for foodstuffs, the EU food business operators and importers are required to ensure food hygiene at all stages in the food chain, as part of the “farm-to-fork” approach. They are legally responsible to comply with the regulation requirements, by ensuring that all imported foods and primary products they produce are safe and protected against contamination arising from air, soil, water, fertilizer, feed, chemicals, worker hygiene, storage, handling, and disposal of wastes (Box 1). Eventually, EFSA members proposed that, while each farm environment is different, the primary objectives for producers should include Good Agricultural Practices (GAP), good hygiene practices (GHPs), and good manufacturing practices (GMPs) [65]. Recently, EFSA identified the generic *E. coli* as suitable for a hygiene criterion that could be considered for validation and verification of GAP and good hygiene practices (GHPs); this allows growers to take appropriate corrective actions [24]. The current European legal framework does not include microbiological criteria applicable at the primary production stage [66].

The guidance document on addressing microbiological risks in fresh fruits and vegetables at primary production through good hygiene offers a detailed description on how to apply the general hygiene requirements and GAP [67].

Box 1 **Annex 1 of Regulation (EC) No. 852/2004. Source: EU Commission [68].**

The general hygiene provisions for primary production and associated operations:

- Keep clean and where necessary, after cleaning, disinfect in an appropriate manner, facilities, equipment, containers, crates, vehicles and vessels
- Ensure hygienic production, transport and storage conditions for, and the cleanliness of plant products
- Use potable or clean water whenever necessary, to prevent contamination
- Ensure that staff handling foodstuffs are in good health and undergo appropriate training
- Prevent, as far as possible, animals and pests from causing contamination
- Store and handle wastes and hazardous substances so as to prevent contamination
- Take account of the results of any relevant analyses carried out on samples taken from plants or other samples that have importance to human health
- Use plant protection products and biocides correctly, as required by the relevant legislation
- Take appropriate remedial action when informed of food safety and hygiene problems identified during official controls

In the US, the FDA (Food and Drug Administration) has been mandated through the FSMA to establish science-based minimal standards for the safe production and harvesting of produce, including minimally-processed fruits and vegetables. The law entails that the FDA develop safe agronomic practices that must be adopted by those who export to the US to improve public health [50].

The FSMA rule also has set of standards that address the same critical areas in the primary production chain, as follows:

- Worker health, hygiene, and training
- Agricultural water, both for production and post-harvest uses
- Biological soil amendments (e.g., compost, manure)
- Domesticated and wild animals
- Equipment, tools, buildings, and sanitation
- Production of sprouts

An example of one of these key requirements (agricultural water) illustrates the rigor of the rule that exporters in the developing countries need to be compliant with. The rule establishes different water quality criteria for water used during crop growing, and during harvest and post-harvest. The criteria are based on the indicator of fecal contamination, i.e., generic *E. coli,* which is adopted as a hygiene criterion by the EFSA [24]. While the rule does not permit detectable generic *E. coli* in the 100 mL water used during or after harvest, it allows a limited amount of *E. coli* in the water used during growing activities. For this, a microbial water quality profile (MWQP) should be developed, based on the results of at least four samples for groundwater sources of agricultural water (e.g., a protected well), and at least 20 samples for surface water sources of agricultural water (e.g., pond, stream, river). The numeric criteria for agricultural water that is intended or likely to contact covered produce (other than sprouts) during growing activities is an MWQP that has the geometric mean (GM) (the average amount of generic *E. coli* in a water source) of ≤126 colony forming units (CFU) of generic *E. coli* per 100 mL, and the statistical threshold value (STV) (*E. coli* levels in adverse conditions, i.e., rainfall) of ≤410 CFU/100 mL generic *E. coli* [69]. If the water exceeds the GM/STV criteria for water that directly come into contact with the edible plants, the FDA rule allows for corrective action as soon as is practical, but not to exceed the following year [69]. 

Because soil amendments of animal origin, such as manure, have been identified as a potential vector for pathogens that may contaminate produce, the FDA rule specifies the interval between application of a biological soil amendment of animal origin and harvest of covered produce. The interval depends on whether that soil amendment has been treated, and on the processes used for the treatment. Standards on detectable amounts of bacteria (including *L. monocytogenes*, *Salmonella* species, fecal coliforms, and *E. coli* O157:H7) have been established to validate these processes. Regarding the application of untreated manure, the rules require a 120-day interval between the application of raw manure for crops in contact with the soil, and 90 days for crops not in contact with the soil [69]. 

Implementation of measures to prevent the introduction of hazards onto covered produce from domesticated and wild animals are obligatory; the same is true for hygienic practices when handling (contacting) covered produce or food-contact surfaces. The equipment and tools that are intended or likely to come in contact with the produce, as well as the building in which growing, harvesting, packing, or holding activities take place, should conform to sanitary standards. With regards to sprouts, the hygienic and sanitary conditions are required during their growing, harvesting, packing, and holding environment, by placing emphasis on testing for *Listeria* species or *L. monocytogenes* through those stages, and testing spent irrigation water from each production batch of sprouts, or the sprouts themselves in the case of *E. coli* O157:H7 and *Salmonella* species.

Both U.S. and EU regulations reflect specific country/regional requirements, but inconsistencies in regulations between countries can negatively impact global trade. To address this concern, international cooperation led to the FAO/WHO Codex Alimentarius standards, and later the World Trade Organization (WTO) with its more specific Sanitary and Phytosanitary (SPS) agreements [17]. The Codex Alimentarius Commission (CAC) was established by the FAO and WHO in 1963. Member countries agree on acceptable standards in a global context. Although it is time-consuming to reach a consensus by all member states, Codex standards have become the benchmarks against which national food measures and regulations are evaluated within the legal parameters of the Uruguay Round Agreements in 1994. The CAC has a great relevance to international trade, as it develops harmonized international food standards, guidelines, and codes of practice, e.g., in relation to specific raw and processed materials, food hygiene, pesticides residues, contaminants and labelling, analysis, and sampling methods, to protect the health of the consumers and ensure balanced trade relationships in food; it also promotes the coordination of all food standards work undertaken by international governmental and non-governmental organizations. For instance, the CAC developed a risk-based *Code of Practice for Fresh Fruits and Vegetables* that addresses GAP and GMPs as a guide to stakeholders to minimize the microbial, chemical, and physical hazards associated with all stages of the production of fresh fruits and vegetables along the food continuum [70]. Many countries adopt these standards because they were generated by international inputs. However, some countries may have similar but not identical standards to Codex. Yet, for trading purposes, they must operate within an international framework of rules and agreements based on scientific principles [20]. 

The final act of the Uruguay Round of Multilateral Trade Negotiations, signed in Marrakech on 15 April 1994, established the WTO Agreement on the Application of Sanitary and Phytosanitary Measures (SPS Agreement). The purpose of this agreement is to ensure the safety of imported food items, which constitutes a major concern for many developed countries. It serves the legitimate purpose of limiting the entry, establishment, and spread of diseases and pests through trade. The SPS measures includes health and hygiene standards or regulations, designed to avoid the spread of animal and plant diseases and epidemics. These measures should be applied to domestically-produced food or local animal and plant diseases, as well as to products coming from other countries. Examples of common sanitary and phytosanitary measures adopted by many countries include importing products from disease-free areas, determining allowable maximum levels of pesticide residues, or the permitted use of only certain additives in food. The agreement emphasizes each country’s freedom and ability to make independent decisions to establish their appropriate level of protection (ALOP) and national standards within the technical regulatory framework, in order to protect human, animal, and plant health, as well as to ensure environmental, wildlife, and human safety. However, the rejection of products according to relevant standards must be based on justification by non-discriminatory scientific input, to ensure that the legal standards and measures adopted by the importing countries are not misused as non-tariff trade barriers, to give unfair advantage to domestically-produced goods. 

**Figure 1:**
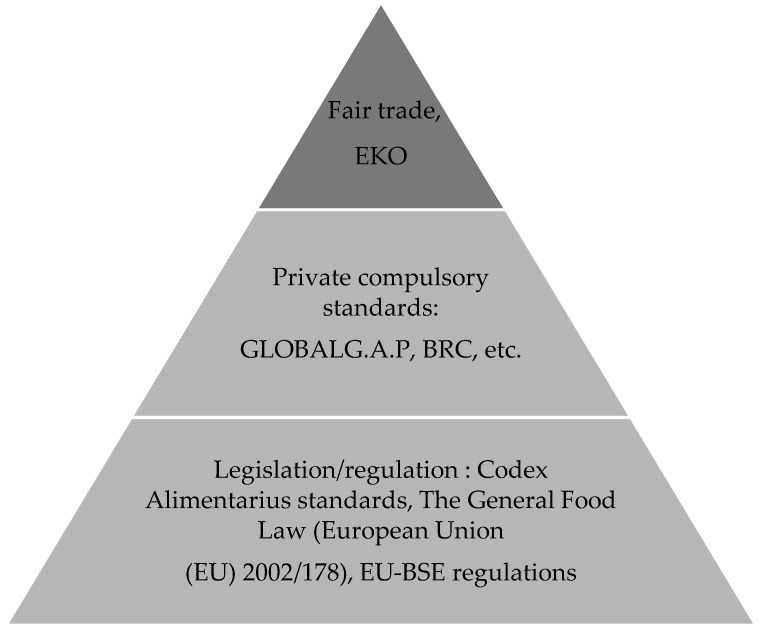
The proliferation of food and agricultural quality standards. Source: Trienekens and Zuurbier [20].

To avoid recalls and bad publicity, the private food industry has placed more emphasis on quality and safety control, the traceability of food products, and environmental issues, by imposing their own specific standards in the value chain [26] (Figure 1). These are not mandated by law, but are market-driven for effective trade throughout the food chain. Although promoted as voluntary, the applications have expanded so significantly in the international markets that many are considered as *de facto* mandatory, such as the GLOBALG.A.P (GAP is an acronym for Good Agricultural Practices) [71]. The stringency and scope of private standards exceed national regulations, since the regulatory environment placed the liability for food safety crisis on the industry and retailers (e.g., the “due diligence” requirements in the UK and the EU) [71]. Therefore, in today’s global market, the world’s largest food retailers are requiring supplier certification in order to participate in the Global Food Safety Initiative (GFSI), which includes the Safe Quality Food Standard (SQF), British Retail Consortium (BRC), International Featured Standards (IFS), Food Safety System Certification (FSSC), GLOBALG.A.P (GAP is an acronym for Good Agricultural Practices), Best Aquaculture Practices (BAP), and Global Red Meat Standard (GRMS). Retailers can also impose related standards, such as are Tesco’s ‘‘Nature’s Choice’’, which puts a number of environmental demands on top of GLOBALG.A.P requirements, eco-labels (i.e., the Dutch EKO-label (EKO is the Dutch word for “eco”. The word “eco” is a prefix mostly relating to ecological or environmental terms)) and fair-trade labels [20]. 

This is similar to the way that many Asian countries recently developed their own national good agricultural practices, such as PhilGAP (Philippines), Q Mark (Thailand), ChinaGAP (China), MyGAP (Malaysia), JGAP (Japan), VietGAP (Vietnam), etc. All those national standards were adapted based on the GLOBALG.A.P requirements. Furthermore, in 2006 and through the secretariat of the Association of Southeast Asian Nations (ASEAN), the ASEAN GAP was launched, as a quality assurance system for ASEAN-region fruits and vegetables. The ASEAN GAP is a standard GAP during the production, harvesting, and post-harvest handling of fresh fruit and vegetables in the ASEAN region. Its objectives are to facilitate the harmonization of national GAP programs in the ASEAN region, facilitate trade regionally and internationally, enhance the safety and quality of fruits and vegetables for consumers, and enhance environmental sustainability [72].

## 4. The Current Status of Food Safety in Developing Countries and the Burden of Compliance Costs

The current requirements in the developed world are well-communicated to stakeholders and promoted with abundant resources to ensure high compliance levels. This is not the case in developing countries, in general. All of these new regulations and private bodies’ standards in high-income countries have a great impact on the trade of potential suppliers from developing countries, as they operate within very different political, institutional, and economic parameters [57]. The MENA countries bordering Europe are in a prime location to export produce to the EU. However, unfortunately, most food control systems in the MENA region are unable to meet the mandated international requirements, due to lack of the required advanced technical and scientific knowledge, political will, and to an unacceptable level of food safety by small-scale farmers and the domestic market [73]. This remains largely unaddressed, despite major reforms being introduced to their national food safety systems, with varying degrees of accomplishments. Many MENA countries have undertaken extensive reviews of their food safety systems in collaboration with WHO, and some have carried out extensive reforms to their national legislation [74]. Over the last decade and more, Egypt, Jordan, Morocco, and Tunisia have reviewed their food safety standards, which were adapted to be in line with the CAC [75,76,77], and recently, the Egyptian Parliament approved the creation of the Egyptian Food Safety Authority [78]. The Sudan and the Syrian Arab Republic have also undergone updates to their food standards and regulations. Development programs were introduced to support the application of GAP in Sudan, especially those involving the safe application of insecticides and fertilizers [79]. 

However, despite the positive policy and regulatory reforms, many developing countries, including those in the MENA region, lack credible institutional mechanisms, meaning the enforcement institutions and water governance are weak, and advocacy is fragmented [80,81,82,83,84,85]. Thus, the agriculture sector is fraught with poor policies for the effective planning of resources, a lack of incentives and training, insufficient knowledge of standards for food safety and quality, and the development of agricultural economies away from efficient resource management [86].

This as a whole has an effect on the performance of the agriculture sector and compliance level of stakeholders. Twenty-seven percent of food exports from Egypt, Jordan, Lebanon, and Syria to the US were rejected in 2001 by the USFDA, due to non-compliance with the US safety measures (such as filth, microbiological contamination, greater-than-permitted levels of pesticide residues, or food additives) [75]. Lately, in 2016, the United Arab of Emirates imposed a ban on fruits and vegetables, including apples, imported from different MENA countries (Egypt, Oman, Yemen, Jordan, and Lebanon) due to high levels of pesticide residues that exceeded the permitted levels according to their own standards [87]. The period of 2002–2011 showed a continuous increment in the RASFF notifications by the EU for products found to be unsuitable for consumption coming from Algeria, Egypt, Jordan, Lebanon, Morocco, Syria, Tunisia, and Turkey. Fruits and vegetables in particular were among the most sensitive exported products, based on the large number of notifications registered [29]. The authors suggested that the rise in alerts indicate increased controls related to regulations and standards. They also presumed that the increment in alerts would continue, if the successive years were to be plotted. 

The European Parliament’s Committee on Agriculture and Rural Development documented cases where selected countries, with whom the EU has agreements concerning trade in food products or is in the process of negotiating such an agreement, did not meet the EU standards (Table 2). These selected countries have many food safety standards, yet they may be out-of-date, or the enforcement is perceived to be inadequate, e.g., the type of pesticides and veterinary drugs allowed, or the associated maximum residue levels are not in line with the EU requirements [88]. In Morocco, for example, compliance with the EU allowable maximum residue limits (MRLs) for pesticides and the associated pre-harvest interval requirements [58] constitute a challenge for exporters. According to the evaluation report of the European Commission, “the authorized uses of plant protection products lead to residues in excess of EU MRLs, and growers are not sufficiently aware of good plant protection practice in this culture” [89]. 

It is very likely that similar shortcomings prevail in MENA countries, given the lack of education, training, and access to GAP in the region, which compromise the safety of fruits and vegetables [85]. The lack of effective water resource management and support of the agricultural sector pose additional threats to water-stressed regions, as farmers revert to insufficiently treated wastewater or sewage-contaminated surface waters for crop irrigation and washing [91,92]. 

Studies reporting on food safety issues in developing countries are very few, but what exists indicates there is widespread contaminated soil; poor agricultural growing methods with the misuse of pesticides, hormones, and fertilizers; and inappropriate post-harvest practices along the food chain, such as the use of untreated wastewater for irrigation and the processing of vegetables [37,73,93,94,95]. The unregulated use of feces-contaminated water for irrigation and the application of untreated manure on fields are classified as primary risk factors; these are largely practiced in Egypt and Lebanon, leading to contamination of the agricultural environment and fresh produce [37,73]. In Egypt, there was a high prevalence of *Salmonella* in strawberries (28%) and lettuce (39%), as well as in soil (42%) and water used for irrigation or washing (42%) [73]. 

Lebanon can be considered as a typical example of many MENA countries struggling to meet international standards. As such, GAP and the integrated pest management are supposed to be promoted through extension services, but there are insufficient staff and resources to carry out the tasks. Public spending for agriculture is highly fragmented in Lebanon, and is scant for food quality and safety programs, under a national budget that has not been updated to cover these areas since 2005 [80,96]. This unfavourable economic condition affects the quality and safety of Lebanese products, which did not progress sufficiently in view of limitations in the enforcement of regulations (e.g., pesticide levels on imported and exported products, or the sanitary condition of transportation vehicles), is the capacity building for technical staff on inspection, Hazard Analysis and Critical Control Points (HACCP), and the infrastructure [97]. *L. monocytogenes* was detected in 20% of the vegetables samples from the fields in Lebanon and after washing in the post-harvest areas; about half of the ready-to-eat vegetables in the fields (51%) contained *S. aureus* at high levels, reaching up to 5 log CFU/g [37]. It is worth noting that the vegetables irrigated with untreated wastewater and polluted river water in Yemen were also found to have *S. aureus* ≥ 5 log CFU/g [98]. Faour-Klingbeil et al. [37] identified critical shortfalls in several areas that constitute major risk factors and contributed to the high levels of pathogens on fresh vegetables. These shortfalls were identified through an assessment of the water usage and sewage treatment, proximity of farm from landfills, sewage treatment, animals/wildlife livestock (access to growing lands), risk assessment during flooding, manure management and storage, manure and municipal biosolids, soils, traceability, field harvesting, storage and transportation, worker health and hygiene, and training. 

Of the findings, the surface water from river streams, mixed with untreated sewage water from nearby villages, was used on fields with no prior treatment. This applies to post-harvest wash water as well. The environmental risk factors were evident, resulting from existing growing lands in proximity to haphazard industry and animal husbandries’ disposals. Growing fields were accessible to animals grazing on the remaining and discarded parts of crops after harvest, to prepare the soils and use animals manure as fertilizers for growing the next crop of parsley. The storage of manure lagoons adjacent to production areas were without provisions to prevent leakage or runoff. The manure’s treatment to eliminate another source of bacterial hazards was dependent on producers’ experience rather than on technical guidelines or experts’ assistance. This is often the case in many non-EU countries, where these operators rely on their own experience, while those in the EU follow standards and guidelines, and have ready access to resources [83].

Clearly, agricultural practices such as those illustrated here pose great risks to health and the environment. They also hinder any rapid progress for trade, in accordance with international standards and WTO [99,100], and lead to the EU banning some national exports [6]. In this regard, in the joint meeting of the United Nations Economic and Social Commission for Western Asia (UNESCWA) and FAO, on setting up a regional Arab Good Agricultural Practices Framework (Arab-GAP), member countries have expressed great concerns over the lack of technical skills and limited support to adopt the international standards. The outcomes revealed the utmost need for the establishment of national GAP standards, to improve the safety of their local production and strengthen the trade with high-value agricultural markets [100]. 

Along with a legal framework, availability of resources is key for the effective participation in trade and compliance to the SPS agreement, as well as to private standards. However, these are commonly deficient in many developing countries [101]. Abiding by legal SPS measures and GLOBALG.A.P impacts the industry, in terms of cost for new inspections, testing facilities and laboratories, certification of inputs and outputs, losses, and delays in shipping products to their final destinations [102]. This increase in cost can hinder exports to the EU region and impact the agricultural sector of importing countries [103]. There is a body of literature on the contribution of non-tariff barriers, such as labels and SPS measures, to the increasing costs of imports or to prohibiting them completely [104], and about how they limit the welfare-enhancing benefits of freer trade [105,106,107]. 

The 2005 economic analysis project in Jordan, Syria, and Egypt is a case in point. Exporters and producers in these countries identified key technical impediments and trade constraints related to SPS, with high costs being the main hurdle to meet SPS requirements [102] (Box 2).

Box 2 **Hurdles to meet Sanitary and Phytosanitary (SPS) requirements. Source: Muaz [102].**

- Serious lack of knowledge about SPS requirements and regulations
- Absence of quality control laboratories in the region to monitor hazards, mainly chemical residues
- High cost of infrastructure needed to meet SPS conditions
- Absence of modern packing and grading facilities
- Lack of inspections to control domestic production and qualified laborers
- Non-existence of local legal bodies responsible for the implementation and monitoring of SPS and other agreements regulations

## 5. Food Safety Standards as a Catalyst to Food Trade

Many researchers do not contend the absolute negative effects of standards, particularly on small holders. They suggest that the standards’ net effect can be advantageous for trade, given the improvements to consumer welfare [108] and the improved well-being of participating farmers [109]. The benefits from the implementation of private standards in the fruits and vegetables chain value on trade between the EU and Tunisia, Morocco, Egypt, and Turkey are tangible [86], but only over a long-term basis [58]. 

**Figure 2:**
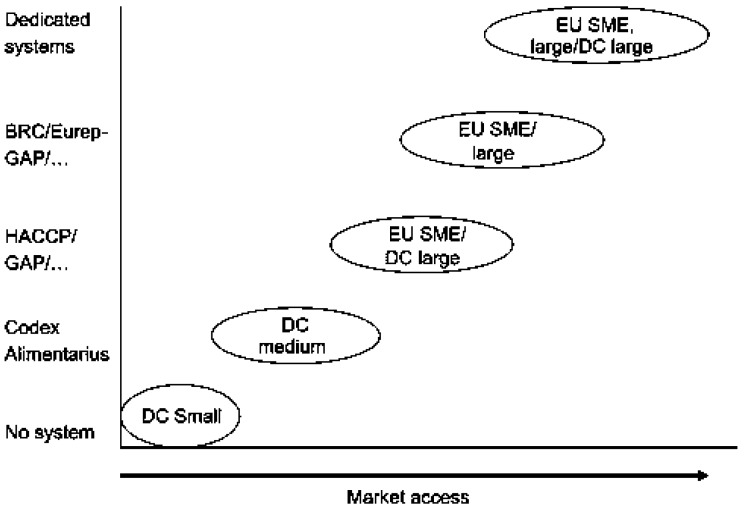
The increasing market access of firms as affected by compliance to standards and firm size. Source: Trienekens and Zuurbier [20]. DC: Developing countries; EU: European Union; SME: small and medium enterprises.

Trienekens and Zuurbier [20] also identified an accelerated growth in the value of exports during the same period when regulatory requirements have been becoming more stringent and complex (Figure 2). The trade activities of small farmers increased, but not to the level observed with the large exporters. The authors noted that although the compliance costs may be significant, they were still small relative to the value of exports or the domestic spillover effects. 

There are many examples where small operators can successfully compete for the export market with vertical coordination, contract farming, and the support of food chain actors, in order to adhere to marketing standards, SPS measures, hygiene standards, and traceability standards [110,111]. In Jordan, the GLOBALG.A.P system is increasingly adopted by farmers who are members of the Jordanian Exporters and Producers Association for Fruits and Vegetables. With funds from the United States Agency for International Development (USAID), the Association is actively engaged in conveying the GLOBALG.A.P requirements to more than 120 members, who represent the major fruit and vegetable exporters in the country. The Association played an outstanding role in boosting the export values of the system by improving the quality and safety of the Jordanian value chain. It enables farmers (members) to meet the regional and the international markets’ requirements [100]; however, such efforts have not been yet adopted for local market production, despite the hazards associated with the use of inadequately treated wastewater. Similarly, in Morocco, farmers with affiliation to cooperatives were able to comply with SPS standards [111]. The members and owners of the packing houses are leaders of the certification movement that is gaining momentum in various horticultural areas in Morocco. As citrus and tomato exporters, they are among those who are implementing a variety of measures, such as labelling, sampling procedures, risk assessment and other food safety measures necessary for certification [112].

In Lebanon, several USAID-funded projects have imposed sets of conditions and requirements for producers to adhere to, but have also proved to enhance the safety of products and their export values, such as Sustainable Agri-Industry in Lebanon (ASAIL) and Agricultural Quality Control and Certification (QCC) programs [113]. These cooperative developments, where they exist, are of great benefit to the companies that participate, primarily for the farmers’ welfare and for the safety and quality of agri-food for local and export markets. Nevertheless, the viability of these initiatives is questionable. Based on examples from countries such as Lebanon, and to a certain extent Jordan, external financing does not provide a long-term solution, particularly under poor institutional framework, national laws, and standards [103]. In addition, these programs do not impact independent farmers and producers with limited means of investments, and do not have as good opportunities for openings to market channels.

For the long term, government engagement is key to the sustainability of programs that encourage better standards for produce and other foods. This should lead to policy development and implementation, economic resources, and incentives, as well as promoting, facilitating, and setting national GAP standards that are consistent with global requirements. The ASEAN GAP can serve a model for the governments interested in promoting good farming practices and improved postharvest handling, with the aim of enhancing the quality of fresh fruits and vegetables, as well as their exports. It is the governments’ commitment and national priority to promote safe and healthy foods, which brought the ASEAN GAP to world recognition [103]. This cooperation between government and industry is illustrated through African examples. 

Case studies in the fisheries sector in Uganda, Kenya, South Africa, and Namibia emphasized the substantial role of an effective and proactive local authority in partnership with the private sector [114,115]. These cases illustrated the influence of standards on the trade performance, through what Henson and Mitullah [115] termed as a “reactive compliance” to the EU food safety and traceability regulations, which denotes efforts to “comply with the standards” or a “reactive exit”, implying an exit from the market. Uganda had initially endured the negative effects of the EU import bans in 1997–2000, which resulted in the closure of many plants and a 20% reduction of work capacity in the remaining plants [114]. By restructuring the entire regulatory and inspection system, upgrading the landing sites for managing the export activities, and the establishment of a “Competent Authority” in charge of fish safety issues and an internationally accredited private laboratory, Uganda recovered from the bans, and all of the companies and their 15 plants are now HACCP compliant. In 2003, their exports increased by 30%. Similarly, Tanzania’s reaction to the ban was proactive, and it became the first country to comply with EU standards, thanks to improvements in governmental monitoring and enforcing regulations [116]. By 2000, the country was able to overcome the ban and recapture its role in the market, and in 2003, Tanzania was able to increase its export share to 60% [58].

## 6. Conclusions 

As many developing and developed countries have discovered, food safety standards and SPS measures can both limit and enhance trade, and the negative and positive impact of standards on their food trade is not easily determined. Nevertheless, a number of case studies and reports have illustrated successful models for the increasing trade values of small farmers who demonstrated compliance to the international requirements necessary for export. To date, compliance has been achieved through the cooperation of downstream actors to these farmers and international aid organizations. This cooperation has, for the most part, not progressed to the next step of a long-term commitment by governments, to enable these suppliers to be independently responsible players in an export food chain. The experiences in six developing countries (Bangladesh, Cambodia, China, the Philippines, Thailand, and Vietnam) established that improvements in the quality requirements in fruit and vegetable production were driven by “reactive compliance” after facing competitiveness problems to access export markets [117]. In those cases, the proactive policies were few; despite a major effort to meet the EU’s Plant Protection Directive (91/414/EEC), and amendments to Japan’s Food Sanitation Law, government ministries of those developing countries had few interactions with the private sector, and a limited role in the dissemination and analysis of the implications of food safety standards on exports. To overcome market constraints, the EU’s directive required donors and intergovernmental organizations are supposed to play a leading role assisting them [117]. 

These six case studies reinforce the need for national governments to foster inter-ministerial coordination and cooperation with the private sector, non-governmental organizations (NGOs), and research institutes, in order to develop strategies for long-term solutions to increase market access. This is crucial, as developed countries are increasingly working on controlling the microbial and chemical risks to reduce contamination and potential foodborne illnesses associated with produce. Governments of importing countries expect a safe food supply, and companies are urged to invest in implementing food safety initiatives. An example is the establishment of the Produce Safety Network (PSN) in the US; this was built to support the efforts of farmers, state regulators, and other key stakeholders, to implement the 2018 Produce Safety Rule as a part of the USFDA Food Safety Modernization Act (FSMA). 

The PSN aims to establish regionally-based policy and regulatory experts throughout the country, to provide technical assistance, conduct outreach and training, participate in work planning, and investigate outbreaks. It also developed a team of experts from two very different FDA offices, whose tasks span beyond domestic control to include foreign inspections [118]. Similarly, in Australia and New Zealand the Fresh Produce Safety Centre (FPSC), an industry-led organization, was established in 2013 to enhance fresh produce food safety, with a new edition of the Guidelines for Fresh Produce Food Safety released in 2015 [119]. These guidelines are designed to assist growers, packers, transporters, wholesalers, retailers, and others involved in the fresh produce supply chain to identify and assess potential food safety hazards, and to help prevent, reduce, or eliminate such hazards, through appropriate practices. However, these efforts are focused on specific aspects of the national produce industry. 

From the limited data available, it is apparent that most MENA countries are not yet able to leverage the safety and quality of the agri-food sector to access the international produce market by understanding and implementing globally-acceptable standards. The independent producers are unable to comply with the necessary private standards to meet stricter safety and legal requirements along the entire food continuum, because of limits in resources, support, and regulatory enforcement by governments. This is compounded by the fact that much of the MENA region is in conflict or in tension with neighbors. For instance, Lebanon’s exporters of fruits and vegetables today are increasingly constrained, and encounter huge losses in view of the regional conflicts and political stress in the neighboring countries, e.g., Israel and Syria. Thus, diverting exports to alternative market channels, such as the EU or the US is important, but constitutes a major challenge because of a higher level of standards for safe products that consumers in these countries demand. To mitigate their impact on national exports, national governments of the MENA countries, particularly countries relying on food exports rather than oil in their economy, should develop sustainable solutions. This requires national governments to draw proactive food safety strategies and health policies that are based on advanced and rigorous scientific studies, to establish national GAP standards and SPS measures consistent with the international standards, guidelines, and recommendations. 

The robust interplay and linkages between the governments, the private sector, and research institutions in developed countries may not be realizable today in many MENA countries, at least not in the short term. One proposed approach to overcome this gap is collaborating with neighboring and regional countries to share national experiences, to establish technical assistance network, and to cooperate in a standard setting, establishing national GAP standards and scientific research.

## Figures and Tables

**Table 1:** Examples of incidences of fresh produce-related outbreaks (2000–2015).

Most Recent Fresh Produce-Related Outbreaks/Country	Number of Cases	Type of Produce/Origin	Remarks
2012–2015: annual outbreaks of Cyclosporiasis (*Cyclospora cayetanensis*) in the US	154 people infected	Cilantro from Mexico	Investigations in July 2015 found that poor hygienic conditions for farm workers were most likely the cause of those outbreaks [6].
2015: *Salmonella* Poona outbreak	767 people infected from 36 states	Cucumbers from Mexico	[51]
2014: *Salmonella* Newport outbreak in 2014	257 people infected in 29 states and the District of Columbia	Cucumbers/unidentified source	The pathogen was assumed to be linked to the application of manure [6].
2012: *S.* Typhimurium and *Salmonella* Newport in 2012	261 people infected in 24 states, 3 deaths and 94 hospitalizations.	Cantaloupes	An inspection found unsanitary conditions in the farm’s processing shed [6].
2011: Major EHEC O104:H4 outbreak in Germany	3000 cases with bloody diarrhea, 852 cases of haemolytic uremic syndrome, and 53 deaths	sprouted fenugreek seeds/traced to shipment of seeds from Egypt to Germany	[52]
2008: *Salmonella* Saintpaul	1442 people infected in 43 states.	Jalapeño and serrano peppers and pepper products (e.g., salsa) from Mexico	Contaminated irrigation water was suspected [6].
2006: Multi-state outbreak of *E. coli* O157:H7	205 sickened and 3 deaths	Spinach	Contaminated fields by swine feces [53].
2005: *S.* Typhimurium in Finland	60 people infected	Lettuce/iceberg imported from Spain	[49]
2005: one outbreak of *E. coli* O157:H7 in Sweden	120 people infected	Lettuce/iceberg	Irrigation from a stream was suspected [54].
2005: one outbreak of *E. coli* O157:H7 in USA	more than 12 sickened	Parsley	[55]
2004: *Salmonella* Thompson in Norway, some cases in Sweden	20 sickened	Rucola/Rocket imported from Italy	[47]
2004: *Salmonella Newport* in UK	375 sickened	Lettuce imported from Spain	[46]
2001: *Listeria*	147 people infected in 28 states and 33 deaths	Cantaloupes	The outbreak was linked to unsanitary conditions at the packing facility on the farm [6].
2000: two *S.* Typhimurium outbreaks in England, Wales, Scotland, Ireland, Germany and Netherlands	392 people infected	Imported lettuce/Iceberg	[48]
2000: *Cyclospora cayetanensis* outbreak in Germany	34 sickened people	Imported lettuce (unidentified)	Probably contaminated through fertilization with human waste or fecal contaminated water used to irrigate crops [56]

**Table 2:** Food safety standards of selected countries, in comparison with the EU as one entity.

Country	Country Standards in Conflict with EU Standards
Brazil	Types of permitted veterinary medicines^1^ Use of ractopamine—an additive banned in the EU—in feedstuffs for pigs Approved genetically modified plants not (yet) approved in the EU Lack of enforcement of compliance with legislation concerning pesticide use, maintenance and sanitary issues in poultry and beef slaughterhouses and processing plants, and traceability of pigs
Morocco	Officially approved self-control systems in vegetable and fruit production are not necessarily based on HACCP Lack of maximum residue levels for residues of pesticides Types of authorized pesticides in Morocco are not allowed in the EU
New Zealand	Identification and registration of sheep is not legally required Lack of enforcement of compliance with legislation concerning record keeping of medical treatment of cows and sheep
Ukraine	Obsolete food safety legislation based on mandatory standards, lack of integrated food control system in line with international standards and WTO, and obsolete enforcement of compliance with legislation
USA	The use of growth promoters in beef cattle^3^ The use of lactic maximum limits and maximum residue limits for mycotoxins and food safety hazards are higher than those in the EU Approved genetically modified plants not (yet) approved in the EU The use of lactic acid as a decontamination step for beef ^2^

Adapted from Van Wagenberg [88]. ^1^ The availability and use of veterinary medicines is much less restricted than in the EU. ^2^ The EU recently approved the use of lactic acid to clean animal carcasses [90]. ^3^ EU bans imports of hormone-treated beef. HACCP: Hazard Analysis and Critical Control Points; WTO: World Trade Organization.

## References

[1] WHO (World Health Organization) (2015). WHO Estimates of the Global Burden of Foodborne Diseases.

[2] Newell D.G., Koopmans M., Verhoef L., Duizer E., Aidara-Kane A., Sprong H. (2010). Food-borne diseases—The challenges of 20 years ago still persist while new ones continue to emerge. Int. J. Food Microbiol..

[3] Wadamori Y., Fam J., Hussain M.A., Gooneratne R., Yildiz F. (2016). Microbiological risk assessment and antibiotic resistance profiling of fresh produce from different soil enrichment systems: A preliminary study. Cogent Food Agric..

[4] Zhu Q., Gooneratne R., Hussain M.A. (2017). Listeria monocytogenes in Fresh Produce: Outbreaks, Prevalence and Contamination Levels. Foods.

[5] European Commission Final Report of an Audit Carried out in Germany from 12 to 22 November 2013 in Order to Evaluate the Official Controls in Primary Production of Food of Non-Animal Origin. https://www.google.de/url?sa=t&rct=j&q=&esrc=s&source=web&cd=1&cad=rja&uact=8&ved=0ahUKEwj5pN_0qMvZAhWCKlAKHfBtAssQFggpMAA&url=http%3A%2F%2Fec.europa.eu%2Ffood%2Ffvo%2Fact_getPDF.cfm%3FPDF_ID%3D11465&usg=AOvVaw15ckxeJO-bpd3IKzzF6I9m.

[6] CSPI (2015). Outbreak Alert!. http://cspinet.org/reports/outbreak-alert-2015.pdf.

[7] Callejón R., Rodríguez-Naranjo M., Ubeda C., Hornedo-Ortega R., Garcia-Parrilla M., Troncoso A. (2015). Reported foodborne outbreaks due to fresh produce in the United States and European Union: Trends and causes. Foodborne Pathog. Dis..

[8] O’Brien T.F. (2002). Emergence, spread, and environmental effect of antimicrobial resistance: How use of an antimicrobial anywhere can increase resistance to any antimicrobial anywhere else. Clin. Infect. Dis..

[9] Gereffi G., Lee J. A global value chain approach to food safety and quality standards. Presented at the Global Health Diplomacy for Chronic Disease Prevention Working Paper Series.

[10] Todd E.C.D., Harris C.K., Knight A.J., Worosz M.R. (2007). Spinach and the media: How we learn about a major outbreak. Food Prot. Trends.

[11] Frank C., Werber D., Cramer J.P., Askar M., Faber M., Heiden M.A., Bernard H., Fruth A., Prager R., Spode A. (2011). Epidemic Profile of shiga-toxin-producing *E. coli* O104:H4 outbreak in Germany. N. Engl. J. Med..

[12] British Broadcasting Corporation (2011). E. coli Cucumber Scare: Spain Angry at German Claims.

[13] Anderson M., Jaykus L.-A., Beaulieu S., Dennis S. (2011). Pathogen-produce pair attribution risk ranking tool to prioritize fresh produce commodity and pathogen combinations for further evaluation (P3ARRT). Food Control.

[14] Food Safety News (2011). EU Ban on Egyptian Fenugreek Seeds Extended. http://www.foodsafetynews.com/2011/10/ban-on-egyptian-fenugreek-seeds-extended/#.Vli8T3arSM8.

[15] Allan J. (2013). The Food Safety Modernization Act—A Series on what is Essential for a Food Professional to Know. Food Prot. Trends.

[16] ANFA (Australia New Zealand Food Authority) (1999). Food Safety Standards—Costs and Benefits.

[17] Boqvist S., Dekker A., Depner K., Grace D., Hueston W., Stärk K., Sternberg Lewerin S. (2014). Contagious animal diseases: The science behind trade policies and standards. Vet. J..

[18] Knowles T., Moody R., McEachern M.G. (2007). European food scares and their impact on EU food policy. Br. Food J..

[19] FAO/WHO (2008). Microbiological Hazards in Fresh Leafy Vegetables and Herbs.

[20] Trienekens J., Zuurbier P. (2008). Quality and safety standards in the food industry, developments and challenges. Int. J. Prod. Econ..

[21] Babu S., Tashmatov A. (2000). Food Policy Reforms in Central Asia: Settings the Research Priorities.

[22] Trench P.C., Narrod C., Roy D., Tiongco M., Fan S., Pandya-Lorch R. (2012). Responding to Health Risks Along the Value Chain. Reshaping Agriculture for Nutrition and Health.

[23] Al-Mazeedi H.M., Abbasa A.B., Al-Jouhara W., Al-Muftya S.A., Al-Mendicara Y.A. (2012). Food Safety Review (FSR) in the State of Kuwait as a part of Arab Gulf Area. Int. J. Food Saf..

[24] EFSA Panel on Biological Hazards (BIOHAZ) (2014). Scientific Opinion on the risk posed by pathogens in food of non-animal origin. Part 2 (*Salmonella* and Norovirus in leafy greens eaten raw as salads). EFSA J..

[25] Food and Agriculture Organization of the United Nations/World Health Organization (FAO/WHO) (2008). Microbiological Hazards in Fresh Fruits and Vegetables.

[26] Jaffee S., Henson S., Luz D.R. Making the Grade: Smallholder Farmers, Emerging Standards, and Development Assistance Programs in Africa—A Research Program Synthesis. https://openknowledge.worldbank.org/handle/10986/2823.

[27] Lee J., Gereffi G., Beauvais J. (2012). Global value chains and agrifood standards: Challenges and possibilities for smallholders in developing countries. Proc. Natl. Acad. Sci. USA.

[28] Food and Agriculture Organization of the United Nations Report on Country Pogramming Framework. http://www.fao.org/tc/policy-support/types-of-support/country-programming-framework/en/.

[29] Taghouti I., Martinez-Gomez V., Alvarez Coque J.M.G. (2015). Exploring EU food safety notifications on agro-food imports: Are Mediterranean partner countries discriminated?. Int. J. Food Agric. Econ..

[30] World Trade Organization (2000). Food Safety and Developing Countries. Agriculture Technology Notes. Rural Development Department (RDV). http://documents.worldbank.org/curated/en/303731468740200070/pdf/multi0page.pdf.

[31] FAOSTAT (2013). Food and Agriculture Organization Corporate Statistical Database. http://faostat3.fao.org/home/index.html#HOME.

[32] U.S. Government Accountability Office (2012). FDA Can Better Oversee Food Imports by Assessing and Leveraging Other Countries’ Oversight Resources.

[33] Keener L., Nicholson-Keener S., Koutchma T. (2014). Harmonization of legislation and regulations to achieve food safety: US and Canada perspective. J. Sci. Food Agric..

[34] Buzby J.C., Unnevehr L.J., Roberts D. (2008). Food Safety and Imports: An Analysis of FDA Food-Related Import Refusal Reports.

[35] Buchholz U., Bernard H., Werber D., Bohmer M.M., Remschmidt C., Wilking H., Kuhne M. (2011). German outbreak of *Escherichia coli* O104:H4 associated with sprouts. N. Engl. J. Med..

[36] Cummings K., Barrett E., Mohle-Boetani J.C., Brooks J.T., Farrar J., Hunt T., Slutsker L. (2001). A multistate outbreak of *Salmonella enterica* serotype Baildon associated with domestic raw tomatoes. Emerg. Infect. Dis..

[37] Faour-Klingbeil D., Murtada M., Kuri V., Todd E. (2016). Understanding the routes of contamination of ready-to-eat vegetables in the Middle East. Food Control.

[38] Franz E., van Diepeningen A.D., de Vos O.J., van Bruggen H.C. (2005). Effects of cattle feeding regimen and soil management type on the fate of *Escherichia coli* O157:H7 and *Salmonella enterica* Serovar Typhimurium in manure-amended soil, and lettuce. Appl. Environ. Microbiol..

[39] Sivapalasingam S., Friedman C.R., Cohen L., Tauxe R.V. (2004). Fresh produce: A growing cause of outbreaks of foodborne illness in the United States, 1973 through 1997. J. Food Prot..

[40] Jung Y., Jang H., Matthews K.R. (2014). Effect of the food production chain from farm practices to vegetable processing on outbreak incidence. Microb. Biotechnol..

[41] Todd E.C., Greig J. (2015). Viruses of foodborne origin: A review. Virus Adapt. Treat..

[42] Strachan N.J.C., Doyle M., Kasuga M., Rotariu O., Odgen I.D. (2005). Dose response modeling of *Escherichia coli* O157 incorporating data from foodborne and environmental outbreaks. Int. J. Food Microbiol..

[43] DeWaal C., GBhuiya F. Outbreaks by the numbers: Fruits and Vegetables 1990–2005. Presented at the International Association for Food Protection 94th Annual Meeting.

[44] Mercanoglu Taban B., Halkman A.K. (2011). Do leafy green vegetables and their ready-to-eat [RTE] salads carry a risk of foodborne pathogens?. Anaerobe.

[45] Powell M., Schlosser W., Ebel E. (2004). Considering the complexity of microbial community dynamics in food safety risk assessment. Int. J. Food Microbiol..

[46] Gillespie I. Outbreak of *Salmonella* Newport Infection in England, Scotland, and Northern Ireland: Association with the Consumption of Lettuce. http://www.eurosurveillance.org/content/10.2807/esw.08.41.02562-en.

[47] Nygård K., Lassen J., Vold L., Andersson Y., Fisher I., Löfdahl S., Aavitsland P. (2008). Outbreak of *Salmonella* Thompson infections linked to imported rucola lettuce. Foodborne Pathog. Dis..

[48] Crook P.D., Aguilera J.F., Threlfall E.J., O’Brien S.J., Sigmundsdóttir G., Wilson D., Widdowson M.A. (2003). A European outbreak of *Salmonella enterica* serotype Typhimurium definitive phage type 204b in 2000. Clin. Microbiol. Infect..

[49] Takkinen J., Nakari U., Johansson T., Niskanen T., Siitonen A., Kuusi M. (2005). A nationwide outbreak of multiresistant *Salmonella* Typhimurium var Copenhagen DT104B infection in Finland due to contaminated lettuce from Spain. Eurosurveillance.

[50] US Food and Drug Administration Safer Fruits and Vegetables: FDA Aims to Set Production Standards. http://driscollfoods.com/wp-content/uploads/2014/02/FDA-Fruits-Vegetables-Guidelines.pdf.

[51] Centers for Disease Control and Prevention Multistate Outbreak of Salmonella Poona Infections Linked to Imported Cucumbers. https://www.cdc.gov/salmonella/poona-09-15/.

[52] Mora A., Herrera A., Lopez C., Blanco J. (2011). Characteristics of the Shiga-toxin-producing enteroaggregative *Escherichia coli* O104:H4 German outbreak strain and of STEC strains isolated in Spain. Int. J. Food Microbiol..

[53] Centers for Disease Control and Prevention (2006). Multistate Outbreaks of Salmonella Infections Associated with Raw Tomatoes Eaten in Restaurants, United States, 2005–2006. MMWR.

[54] Söderström A., Lindberg A., Andersson Y. (2005). EHEC O157 outbreak in Sweden from locally produced lettuce. Eurosurveillance.

[55] FSnet E. coli Infections Traced to Contaminated Parsley. http://archives.foodsafety.ksu.edu/fsnet/2005/10-2005/fsnet_oct_31.htm#story.

[56] Döller P., Dietrich K., Filipp N., Brockmann S., Dreweck C., Vonthein R., Wiedenmann A. (2002). Cyclosporiasis Outbreak in Germany Associated with the Consumption of Salad. Emerg. Infect. Dis..

[57] Grote U., Kirchhoff S. (2001). Environmental and Food Safety Standards in the Context of Trade Liberalization: Issues and Options.

[58] Frohberg K., Grote U., Winter E. EU food safety standards, traceability and other regulations: A growing trade barrier to developing countries’ exports?. Proceedings of the International Association of Agricultural Economists Conference.

[59] Gil M.I., Selma M.V., Suslow T., Jacxsens L., Uyttendaele M., Allende A. (2015). Pre- and postharvest preventive measures and intervention strategies to control microbial food safety hazards of fresh leafy vegetables. Crit. Rev. Food Sci. Nutr..

[60] Shames L. (2008). Food Safety: Improvements Needed in FDA Oversight of Fresh Produce.

[61] Da Silva Felício M.T., Hald T., Liebana E., Allende A., Hugas M., Nguyen-The C., McLauchlin J. (2015). Risk Ranking of Pathogens in Ready-to-Eat Unprocessed Foods of Non-Animal Origin (FoNAO) in the EU: Initial Evaluation using Outbreak Data (2007–2011). Int. J. Food Microbiol..

[62] EFSA Panel on Biological Hazards (2014). Scientific opinion on the risk posed by pathogens in food of non-animal origin. Part 2 (*Salmonella* in melons). EFSA J..

[63] EFSA Panel on Biological Hazards (2014). Scientific opinion on the risk posed by pathogens in food of non-animal origin. Part 2 (*Salmonella* and Norovirus in berries). EFSA J..

[64] EFSA Panel on Biological Hazards (2011). Scientific Opinion on the risk posed by Shiga toxin-producing *Escherichia coli* (STEC) and other pathogenic bacteria in seeds and sprouted seeds. EFSA J..

[65] EFSA Panel on Biological Hazards (2013). Scientific opinion on the risk posed by pathogens in food of non-animal origin. Part 1 (outbreak data analysis and risk ranking of food/pathogen combinations). EFSA J..

[66] Allende A., Dattab A.R., Smith W.A., Adonis R., MacKay A., Adell D. (2017). Implications of new legislation (US FSMA) and guidelines (EC) on the establishment of management systems for agriculture water. Food Microbiol..

[67] Coulon S. Commission Notice on Guidance Document on Addressing Microbiological Risks in Fresh Fruits and Vegetables at Primary Production through Good Hygiene (2017/C 163/01). Proceedings of the 3rd meetings if the CIS Ad-hoc Task-Group on water reuse.

[68] EU Commission (2004). Regulation (EC) No. 852/2004 of the European Parliament and of the Council. http://eur-lex.europa.eu/legal-content/EN/TXT/PDF/?uri=CELEX:32004R0852&from=EN.

[69] U.S. Department of Health & Human Services/U.S. Food & Drug Administration (2015). Federal Register/Vol. 80, No. 228/Friday, 27 November 2015/Rules and Regulations. Standards for the Growing, Harvesting, Packing, and Holding of Produce for Human Consumption. https://www.gpo.gov/fdsys/pkg/FR-2015-11-27/pdf/2015-28159.pdf.

[70] CAC/RCP 53 (2003). Code of Hygienic Practice for Fresh Fruits and Vegetables. www.fao.org/input/download/standards/10200/CXP_053e_2013.pdf.

[71] Liu P. Private Standards in International Trade: Issues and Opportunities. Presented at the WTO’s Workshop on Environment-Related Private Standards Certification and Labelling Requirements.

[72] Layese G.F. Asean good agricultural practices Food Safety Module. https://www.unece.org/fileadmin/DAM/trade/agr/promotion/2011_Thailand/ASEAN-GAP_FoodSafetyModule.pdf.

[73] Uyttendaele M., Moneim A., Ceuppens S., Tahan F. (2014). Microbiological Safety of Strawberries and Lettuce for Domestic Consumption in Egypt. J. Food Process. Technol..

[74] United States Trade Representative (2007). Qatar Trade Estimate Report on Foreign Trade Barriers. https://ustr.gov/archive/Document_Library/Reports_Publications/2007/2007_NTE_Report/Section_Index.html.

[75] CSPI Food Safety around the World. https://www.cspinet.org/new/pdf/global.pdf.

[76] FAO/WHO FAO/WHO Regional Meeting on Food Safety for the Near East. www.fao.org/tempref/docrep/fao/Meeting/009/y6024e/y6024e00.pdf.

[77] Food and Agriculture Organization of the United Nations (2004). Food Safety and International Trade in the Near East Region.

[78] Official Gazette (2017). Law No. 1/2017 on Promulgating National Food Safety Authority Law. http://nfsa.gov.eg/Images/App_PP/DeskTop/App_Web/1/MyWebMedia/PDF/NFSA%20Law-English.pdf.

[79] Food and Agriculture Organization of the United Nations (2015). Country Programming Framework for Sudan Plan of Action (2015-2019): Resilient Livelihoods for Sustainable Agriculture, Food Security and Nutrition.

[80] Farajalla N., Kerkezian S., Farhat Z., El Hajj R., Matta M. (2015). The Way Forward to Safeguard Water in Lebanon: Naional Water Intergrity Risk Assessment.

[81] Shouq K. Agriculture Market Report in the MENA Region. https://de.slideshare.net/princesssania7/agriculture-market-report-in-the-mena-region.

[82] Abdel-Dayem S. (2011). Water Quality Management in Egypt. Int. J. Water Resour. Dev..

[83] Kirezieva K., Luning P.A., Jacxsens L., Allende A., Johannessen G.S., Tondo E.C., van Boekel M.A.J.S. (2015). Factors affecting the status of food safety management systems in the global fresh produce chain. Food Control.

[84] Loconto A.M., Dankers C. (2014). Impact of International Voluntary Standards on Smallholder Market Participation in Developing Countries: A Review of the Literature.

[85] Kader A.A., Kitinoja L., Hussein A.M., Abdin O., Jabarin A., Sidahmed A.E. (2012). Role of Agro-Industry in Reducing Food Losses in the Middle East and North Africa Region.

[86] Milli S. (2017). Benchmarking Agri-Food Value Chain Performance Factors in South Mediterranean Countries. Proc. Syst. Dyn. Innov. Food Netw..

[87] Namrouqa H. (2017). UAE Says Vegetable Ban ‘Precautionary’ as Jordan Reaffirms Commitment to International Standards. http://www.jordantimes.com/news/local/uae-says-vegetable-ban-precautionary-jordan-reaffirms-commitment-international-standards.

[88] Van Wagenberg C.P.A., Brouwer F.M., Hoste R., Rau M.L. (2012). Comparative Analysis of EU Standards in Food Safety, Environment, Animal Welfare and Other Non-Trade Concerns with Some Selected Countries.

[89] European Commission Health and Consumers Directorate General Final Report of a Mission Carried out in Morocco. http://ec.europa.eu/food/fvo/act_getPDF.cfm?PDF_ID=8799.

[90] EFSA Panel on BIOHAZ (2014). Scientific Opinion on the evaluation of the safety and efficacy of peroxyacetic acid solutions for reduction of pathogens on poultry carcasses and meat. EFSA J..

[91] Bouchrif B., Paglietti B., Murgia M., Piana A., Cohen N., Ennaji M., Timinouni M. (2009). Prevalence and antibiotic-resistance of *Salmonella* isolated from food in Morocco. J. Infect. Dev. Ctries.

[92] Zyadin A. (2013). Water Shortage in MENA Region: An Interdisciplinary Overview and a Suite of Practical Solutions. J. WARP.

[93] Aiat Melloul A., Hassani L. (1999). *Salmonella* infection in children from the wastewater-spreading zone of Marrakesh city (Morocco). J. Appl. Microbiol..

[94] Hanjra M.A., Blackwell J., Carr G., Zhang F., Jackson T.M. (2011). Wastewater irrigation and environmental health: Implications for water governance and public policy. Int. J. Hyg. Environ. Health.

[95] Sadik A.-K., El-Solh M., Saab N. (2014). Arab Environment∙7 Food Security Challenges and Prospects.

[96] Mhanna M. Mission Final Report on Fruit and Vegetable Good Agricultural Practices in Lebanon: Strengthening Production & Marketing of Lebanese Agricultural Products (Project GCP/LEB/021/ITA). https://www.unescwa.org/events/arab-gap-consultation-1st.

[97] Ministry of Agriculture Ministry of Agriculture Strategy 2015–2019. faolex.fao.org/docs/pdf/leb149670.pdf.

[98] Al-Jaboobi M., Tijane M., El-Ariqi S., Bouksaim M. (2013). Physicochemical and Microbiological Evaluation of Irrigated Vegetables with Wastewater “Yemen”. Middle-East J. Sci. Res..

[99] Gordon A. (2015). Food Safety and Quality Systems in Developing Countries Volume One Export Challenges and Implementation Strategies.

[100] ESCWA Scope and Setting up of an Arab—Good Agricultural Practices Framework (Arab-GAP). https://www.unescwa.org/events/arab-gap-consultation-1st.

[101] Rahmat S., Cheong C.B., Hamid M.S.R.B.A. (2016). Challenges of Developing Countries in Complying Quality and Enhancing Standards in Food Industries. Procedia Soc. Behav. Sci..

[102] Muaz S. (2005). An Economic Analysis of Food Safety Standards and its Implication on Agricultural Trade in the Context of EU-MED Partnership “The Case of SPS Standards and EUREPGAP Requirements”.

[103] United Nations Challenges and Opportunities Arising from Private Standards on Food Safety and Environment for Exporters of Fresh Fruit and Vegetables in Asia: Experiences of Malaysia, Thailand and Vietnam. Unctad.org/en/Docs/ditcted20076_en.pdf.

[104] Roberts D., Krissoff B. Regulatory Barriers in International Horticultural Markets. http://usda.mannlib.cornell.edu/usda/ers/WRS/2000s/2004/WRS-01-09-2004_Special_Report.pdf.

[105] Henson S., Masakure O., Cranfield J. (2011). Do Fresh Produce Exporters in Sub-Saharan Africa Benefit from GlobalGAP Certification?. World Dev..

[106] Henson S. (2007). The Role of Public and Private Standards in Regulating International Food Markets. J. Int. Agric. Trade Dev..

[107] Wilson N.L.W., Anton J. (2006). Combining Risk Assessment and Economics in Managing a Sanitary-Phytosanitary Risk American. J. Agric. Econ..

[108] Wilson N.L.W. (2017). Labels, Food Safety, and International Trade.

[109] Minten B., Randrianarison L., Swinnen J.F.M. (2009). Global Retail Chains and Poor Farmers: Evidence from Madagascar. World Dev..

[110] Kleinwechter U., Grethe H. The Adoption of the Eurepgap Standard by Mango Exporters in Piura, Peru. Presented at the International Association of Agricultural Economists.

[111] Maertens M., Swinnen J. (2009). Trade, Standards, and Poverty: Evidence from Senegal. World Dev..

[112] Aloui O., Kenny L. (2005). The Cost of Compliance with SPS Standards for Moroccan Exports: A Case Study.

[113] ACDI/VOCA (2013). ACDI/VOCA Lebanon. http://www.acdivoca.org/news/by-country/lebanon/.

[114] Ponte S. (2007). Bans, Tests and Alchemy: Food Safety Standards and the Ugandan Fish Export Industry. Agric. Hum. Values.

[115] Henson S., Mitullah W. (2004). Kenya Exports of Nile Perch: Impact of Food Safety Standards on an Export-Oriented Supply Chain.

[116] Musonda F.M., Mbowe W. The Impact of Implementing SPS and TBT Agreements: The Case of Fish Exports to European Union by Tanzania. http://www.tzonline.org/pdf/theimpactofimplementingspsandtbt.pdf.

[117] United Nations Conference on Trade and Development (2007). Food Safety and Environmental Requirements in Export Markets—Friend or Foe for Producers of Fruit and Vegetables in Asian Developing Countries?.

[118] US Food and Drug Administration The Produce Safety Network: Supporting Regulators and Growers across the Country. https://www.fda.gov/Food/GuidanceRegulation/FSMA/ucm579382.htm?utm_campaign=CU%3A%20FDA%20Network%20Takes%20a%20New%20Approach%20to%20Produce%20Safety&utm_medium=email&utm_source=Eloqua.

[119] Fresh Produce Safety Center Guidelines for Fresh Produce Safety. https://producesafetycentreanz.files.wordpress.com/2015/10/guidelines_081015_screen.pdf.

